# SARS‐CoV‐2 viral dynamic modeling to inform model selection and timing and efficacy of antiviral therapy

**DOI:** 10.1002/psp4.13022

**Published:** 2023-08-21

**Authors:** Shengyuan Zhang, Akosua A. Agyeman, Christoforos Hadjichrysanthou, Joseph F. Standing

**Affiliations:** ^1^ Department of Pharmaceutics, School of Pharmacy University College London London UK; ^2^ Infection, Immunity and Inflammation Research and Teaching Department, Great Ormond Street Institute of Child Health University College London London UK; ^3^ Department of Mathematics University of Sussex Brighton UK; ^4^ Department of Infectious Disease Epidemiology, School of Public Health Imperial College London London UK

## Abstract

Mathematical models of viral dynamics have been reported to describe adequately the dynamic changes of severe acute respiratory syndrome‐coronavirus 2 viral load within an individual host. In this study, eight published viral dynamic models were assessed, and model selection was performed. Viral load data were collected from a community surveillance study, including 2155 measurements from 162 patients (124 household and 38 non‐household contacts). An extended version of the target‐cell limited model that includes an eclipse phase and an immune response component that enhances viral clearance described best the data. In general, the parameter estimates showed good precision (relative standard error <10), apart from the death rate of infected cells. The parameter estimates were used to simulate the outcomes of a clinical trial of the antiviral tixagevimab‐cilgavimab, a monoclonal antibody combination which blocks infection of the target cells by neutralizing the virus. The simulated outcome of the effectiveness of the antiviral therapy in controlling viral replication was in a good agreement with the clinical trial data. Early treatment with high antiviral efficacy is important for desired therapeutic outcome.

**Study Highlights**

**WHAT IS THE CURRENT KNOWLEDGE ON THE TOPIC?**

Various mechanistic and empirical models have been suggested for severe acute respiratory syndrome‐coronavirus 2 viral load data but these have not been systematically compared on data including the whole time course of viral trajectory.

**WHAT QUESTION DID THIS STUDY ADDRESS?**

The research performed model selection based on a dataset including the whole infection time course to explore the importance of viral inhibition and timing of an antiviral therapy. Simulated outcome was compared to the published clinical data.

**WHAT DOES THIS STUDY ADD TO OUR KNOWLEDGE?**

Simulation based on the selected model shows a good agreement with the clinical trial data. Early intervention and high efficacy are important for the therapeutic outcome of antiviral drugs in face of new variants.

**HOW MIGHT THIS CHANGE DRUG DISCOVERY, DEVELOPMENT, AND/OR THERAPEUTICS?**

The suggested model can predict viral dynamics within an individual. The viral dynamic model selection method could be applied for simulation of viral trajectories in antiviral trial design. Such models can be used for the assessment of potential prophylactic and therapeutic treatments in drug development.

## INTRODUCTION

Coronavirus disease 2019 (COVID‐19), a respiratory disease caused by severe acute respiratory syndrome‐coronavirus 2 (SARS‐CoV‐2) virus infection, has caused more than 6.7 million deaths globally, including over 200,000 deaths in the United Kingdom alone as of January 8, 2023.^1^ Although various vaccines have been developed, the risk of infection and re‐infection increases with time since receipt of vaccination.^2^ For example, the effectiveness of the ChAdOx1‐S vaccine against symptomatic infection with Delta variant declined from 64.8% the first week after full vaccination to 44.3% after 20 weeks.^2^ In addition, vaccines are less effective against emerging variants of concern.^2^, ^3^, ^4^ Considering the Omicron variant, the titers of neutralizing antibodies elicited by standard two‐dose vaccines are notably reduced compared to the other variants.^5^, ^6^ Moreover, the elderly and immunocompromised individuals are more vulnerable to the development of severe disease.^7^, ^8^ Thus, antiviral therapies are still needed to prevent severe infections and reduce the risk of death in the most vulnerable population.

From the experience of past epidemics, such as influenza and the Middle East respiratory syndrome, viral dynamic models can describe the change in viral load over time and the impact of antiviral therapies.^9^, ^10^ The target‐cell limited (TCL) model, for example, is a classic model that has been used to describe respiratory viral infections, such as influenza and hepatitis C, and was recently applied for the description of SARS CoV‐2 viral dynamics.^9^, ^10^, ^11^ This model has been extended to incorporate the lag time between the cell infection and virus production, and innate or adaptive immune response that increase viral clearance and/or decrease viral production.^12^, ^13^, ^14^ Goyal et al.^15^ introduced a two‐stage immune response, an innate immune system that eliminates infected cells, and a slower acquired cytolytic response with a maximum cell‐killing rate controlled by effector cells. Whereas the models have been shown to perform well in describing different viral load data in different cases, the comparison of their performance on the same dataset is limited.^16^

The TCL model has also been extended to include the impact of antiviral therapies on controlling viral dynamics.^10^, ^17^, ^18^ Such models have become valuable tools in the design of clinical trials and the assessment of candidate therapies as they facilitate, for example, the selection of antiviral drugs and dosing timing. Gonçalves et al.^19^ showed that the required efficacy of SARS‐CoV‐2 treatment could reduce from over 90% to 60% if treatment administration is initiated before symptom onset, suggesting the importance of timing in antiviral treatment. Similar conclusion was noted by Goyal et al.,^15^ emphasizing the significance of potency and timing of antiviral therapies. However, both studies were conducted at the beginning of the pandemic. Considering the change in viral load kinetics of different variants, the effect of antiviral therapies needs re‐assessment.

A combination of SARS‐CoV‐2 neutralizing monoclonal antibodies, tixagevimab and cilgavimab (Evusheld/AZD7442, Catalent), has showed in vitro neutralization activity against the original strain and some variants of concern, including the Delta variant.^20^ The antibodies are derived from B cells isolated from patients infected by SARS‐CoV‐2.^21^ They can block cell entry and neutralize the virus by binding to distinct epitopes on the spike protein receptor‐binding domain.^20^, ^21^ In a clinical trial, its effective antiviral activity was proved, with a relative risk reduction of 50.5% for patients developing severe COVID‐19 or death.^22^

In this study, eight viral dynamic models were fitted to the same dataset. Their performance was assessed and compared with standard model diagnostics and simulation‐based model diagnostic methods. Possible covariates, such as age and vaccination status, were tested. The best‐fit model was used to incorporate mode of action of tixagevimab‐cilgavimab and assess its efficacy. The effect of different treatment administration times and viral inhibition efficacies were tested with in silico experiments, and the simulated results were compared with the reported clinical data.

## METHODS

### Study data

#### Data source

The SARS‐CoV‐2 viral load data were taken from a longitudinal cohort study of community transmission in the United Kingdom, the Assessment of Transmission and Contagiousness of COVID‐19 in Contacts (ATACCC) study.^23^ Household and non‐household contacts were recruited within 5 days since index case symptom onset. Upper respiratory tract samples were collected for the quantification of viral load and identification of the variant. Two waves were captured during the study, a pre‐alpha and alpha wave and a delta wave. Patients did not receive any antiviral treatment during the study. Variants were confirmed by whole genome sequencing, whereas the vaccination status was obtained from the UK National Immunization Management System, GP records as well as self‐report. Unvaccinated was defined as no vaccine dose received at least 7 days before enrollment. If one dose was received at least 7 days before enrollment, the participant was defined as partially vaccinated, and fully vaccinated indicated receipt of two doses at least 7 days before enrollment.

#### Viral load

Quantitative reverse transcription polymerase chain reaction was used to quantify the viral load in the sample. ORF1ab cycle threshold (Ct) values were reported in the dataset. The values were converted into viral RNA copies per mL using the equation below^23^:

(1) $$\text{viral}\;\mathrm{RNA}\;\text{copies}\;\mathrm{per}\;\mathrm{mL}=133.3333\times e^{\frac{\left(37.933-\mathrm{Ct}\right)}{1.418}}.$$

A maximum Ct value of 40 was regarded as the limit of detection (LOD).

#### Time since contact with an infectious individual

Mean days from virus exposure to viral load peak was calculated to be 7 days, for infectious non‐household contacts. This time was then used to calculate the contact time for household contacts, which is not reported in the dataset.

#### The tixagevimab‐cilgavimab clinical trial and simulation

The study was a phase III, randomized, double‐blind, placebo‐controlled study.^22^ Non‐hospitalized adult patients without any vaccination were recruited and randomly assigned to the tixagevimab‐cilgavimab or placebo group (413 in the drug group and 412 in the placebo group). The drug was given 7 days or less since symptom onset through intramuscular administration. Viral sequencing indicated that the majority (60%) of the patients were infected with the alpha variant, and the rest of the patients were infected with the gamma, delta, lambda, mu, and beta variants (accounted for 20%, 15%, 5%, 1%, and <1% of the patients, respectively).

Due to the limitation of the computing power, 112 patients aged from 18 to 78 years were simulated in each group (the tixagevimab‐cilgavimab group or the placebo group). The parameters used for simulation were extracted from the model that was fitted to unvaccinated patients. As it was in the source data, the simulation included the pre‐alpha, alpha, and delta variants.^23^ The viral inhibition efficacy of the drug was assumed to be 99% and the limit of quantification (LOQ) was set to 3.384 log_10_ copies per mL as reported in literature.^22^

### Statistical analysis

#### Viral dynamic models

**FIGURE 1:**
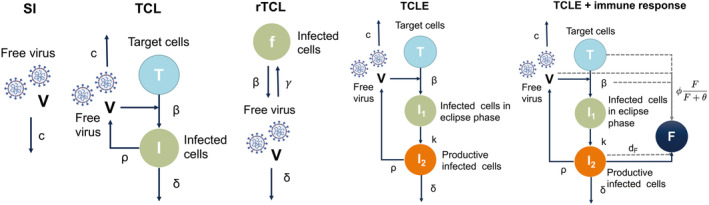
Schematic diagrams of the SI model, TCL model and its extended versions. SI, slope‐intercept; TCL, target‐cell limited.

Schematic diagrams of the slope‐intercept (SI) model and different versions of the TCL model are shown in Figure 1. The SI model is the simplest model in which the viral load within a patient (*V*) is cleared at rate c. In the TCL model, uninfected target cells (T) get infected at a constant rate β in the presence of virus particles (V). Infected target cells (I) shed virus at the production rate ρ, and die at rate δ. The free virus particles are cleared at rate c. The TCL model is unidentifiable because some of the parameters, such as β and ρ, are difficult to measure independently.^24^ To deal partly with the problem, a quasi‐steady‐state was assumed under the assumption that V is changing much faster than I. We call this the reduced TCL (rTCL) model. In the rTCL model, the number of targeted cells (*T*) is replaced by the fraction of uninfected cells, *f*.^10^

The TCL model with an eclipse phase (TCLE model) was also tested.^25^, ^26^ The model assumes that the infected cells are first in an eclipse phase ($I_{1}$; not yet producing virus) and then become productive infected cells ($I_{2}$) at a rate k. $I_{2}$ shed virus at the production rate ρ, and die at rate δ.

The TCLE model, as well as the previous models, does not explicitly describe the role of immune responses. A compartment representing the effect of immune responses F was introduced to the TCLE model. F is assumed to be produced at a rate q by productively infected cells ($I_{2}$) and eliminated at a rate $d_{F}.$ Four additional models describing different effects of innate and adaptive immune response were fitted to the data, including (1) increasing the number of cells refractory to infection, (2) blocking cell infection, (3) increasing viral clearance, and (4) promoting cytotoxicity.^9^, ^12^, ^13^, ^25^, ^27^, ^28^ The effects of F, defined as $\mathrm{\phi F}/\left(F+\theta \right)$, followed a maximum effect relationship, where ϕ represents the maximal effect of immune response and θ is the level of F needed to get half of the maximal effect. Differential equations of the tested models are shown in Supplementary material S1.

#### Parameter estimation and model evaluation

The model was fitted to the viral load data using nonlinear mixed‐effect modeling in the following form:

(2) $$\begin{array}{c} Y_{\mathrm{ij}}=\log M\left(t_{\mathrm{ij}},\varphi_{i}\right)+\varepsilon_{\mathrm{ij}}. \end{array}$$

$Y_{\mathrm{ij}}$ is the viral load in log scale of subject i at time $t_{\mathrm{ij}}$. *M* is the model used. $\varphi_{i}$ are the model parameters, and $\varepsilon_{\mathrm{ij}}$ is the additive residual error, which is assumed to follow a normal distribution.

Parameter estimation was performed by maximum likelihood with the stochastic approximation expectation maximization. R software (version 4.2.1), RxODE package (version 1.1.5), and *nlmixr* package (version 2.0.7) were used in the modeling work.

Graphic model evaluation was undertaken by basic goodness‐of‐fit (GOF) plots and visual predictive checks (VPCs).^29^ GOF plots included observed viral load versus predicted population average and individual viral load in natural log scale. Normalized Prediction Distribution Errors were also plotted with time since contact and predicted viral load. The ideal plot should follow a $N\left(0,1\right)$ distribution without any trend. The VPCs compared the 5th, 50th and 95th percentile of the observed viral load with 90% prediction intervals of the simulated values. Viral loads reported below the LOD were included as censored observations and the probability of the data being below the LOD was calculated. Bayesian information criterion (BIC) were also calculated to compare the model fit.

#### Assumptions on parameters and initial condition

Some unidentifiable parameters were fixed to values reported in the literature. $d_{F}$ was fixed to 0.4 $d^{-1}.$ ^14^ The initial number of target susceptible epithelial cells ($T_{0}$) was fixed to $1.33\times 10^{5}$ cells per mL.^14^ At t=0, $T=T_{0}$; $I_{1}=0$; $I_{2}=1/30$; and $V=V_{0}.$ ^14^

#### Covariate analysis

A covariate analysis considering age, vaccination status, contact type (household/non‐household), type of variant, and symptomatic condition was undertaken. Initial selection of data was performed with graphic inspection, and then numerically tested. Covariates showing level of significance p < 0.05 in the likelihood ratio test were included in the final model.

### Simulations for antiviral therapies

The best fit model and its estimated parameters were used to simulate the effect of antiviral therapies. The antiviral effect was measured with change in viral load area under the curve (AUC) and the proportion of target cells remaining uninfected.^10^ Viral load data were simulated up to day 28, when the viral load becomes below the LOD.^30^ Time of the initiation of treatment ($t^{*}$) and antiviral efficacy (0≤η≤1) were varied. The effect of antiviral therapy was defined as $1-\mathrm{\eta H}\left(t\right)$, where $H\left(t\right)$ is the treatment indicator defined as $H\left(t\right)=0$ if $t<t^{*}$; otherwise, $H\left(t\right)=1$. The drug neutralizes the viruses before they enter the host cells, preventing the infection of susceptible cells. This is described by the reduction of the rate at which cells become infected when they interact with infectious virus particles, β. The model used for the simulation is as follows:

(3) $$\begin{array}{c} \frac{dT}{dt}=-\left(1-\eta H\left(t\right)\right)\beta \mathrm{VT} \end{array}$$

(4) $$\begin{array}{c} \frac{dI_{1}}{dt}=\left(1-\eta H\left(t\right)\right)\beta \mathrm{VT}-kI_{1} \end{array}$$

(5) $$\begin{array}{c} \frac{dI_{2}}{dt}=kI_{1}-\delta I_{2} \end{array}$$

(6) $$\begin{array}{c} \frac{dV}{dt}=\rho I_{2}-\mathrm{cV}-\phi \frac{F}{F+\theta }V-\left(1-\eta H\left(t\right)\right)\beta \mathrm{VT} \end{array}$$

(7) $$\begin{array}{c} \frac{dF}{dt}=I_{2}-d_{F}F \end{array}$$

## RESULTS

### Baseline characteristics

One hundred sixty‐two patients were included in the present study with a total of 2155 observations. Baseline characteristics are summarized in Table 1. In the selected dataset, patients aged 7 to 78 years were recruited, with the median being 36 years. The majority of patients were 18 to 48 years (56.17%), whereas five (3.09%) patients were aged greater than or equal to 65 years. As per contact type, there was 124 household contacts (76.54%) and 38 non‐household contacts (38%). About 70.37% of the patients did not receive any treatment at least 7 days before enrollment, and 23.46% of the patients were fully vaccinated at least 7 days before enrollment. Genome sequencing confirmed that 70 patients were infected by the delta variant (43.21%), 50 infected by the pre‐alpha variant (30.86%), and 42 by the alpha variant (25.93%). The symptomatic status of 146 cases was available; over half was reported as symptomatic (55.56%; *n* = 90).

**TABLE 1:** Demographic characteristics of the 162 patients involved in the study.

	*N* (%)
Total number of patients	162 (100)
Age, years	
<18	24 (14.81)
18–48	91 (56.17)
49–64	42 (25.93)
≥65	5 (3.09)
Contact type	
Household	124 (76.54)
Non‐household	38 (23.46)
Vaccination	
Unvaccinated	114 (70.37)
Partially vaccinated	10 (6.17)
Fully vaccinated	38 (23.46)
Variant	
Pre‐alpha	50 (30.86)
Alpha	42 (25.93)
Delta	70 (43.21)
Symptomatic	
Symptomatic	90 (55.56)
Asymptomatic	56 (34.57)

### Viral dynamic modeling

#### Structural model selection

Compared with SI, TCL, and rTCL models, TCLE model showed the lowest BIC, indicating a better fit to the data (Table 2). As a result, the TCLE model was chosen as the basic model that has been used to incorporate the effects of immune response against viral infection. The TCLE model with immune response that increases viral clearance (M7 model, Table 2) showed the best fit to the data.

**TABLE 2:** Comparison of the viral dynamic models.

Model	Description	BIC
M1	SI	13,573
M2	TCL	12,966
M3	rTCL	12,351
M4	TCLE	12,203
M5	TCLE + increasing cell refractory	12,333
M6	TCLE + blocking cell infection	12,290
M7	TCLE + increasing viral clearance	12,182
M8	TCLE + promoting cytotoxicity	12,251

Abbreviations: BIC, Bayesian information criterion, rTCL, reduced target‐cell limited; SI, slope‐intercept; TCL, target‐cell limited; TCLE, TCL model with an eclipse phase.

**FIGURE 2:**
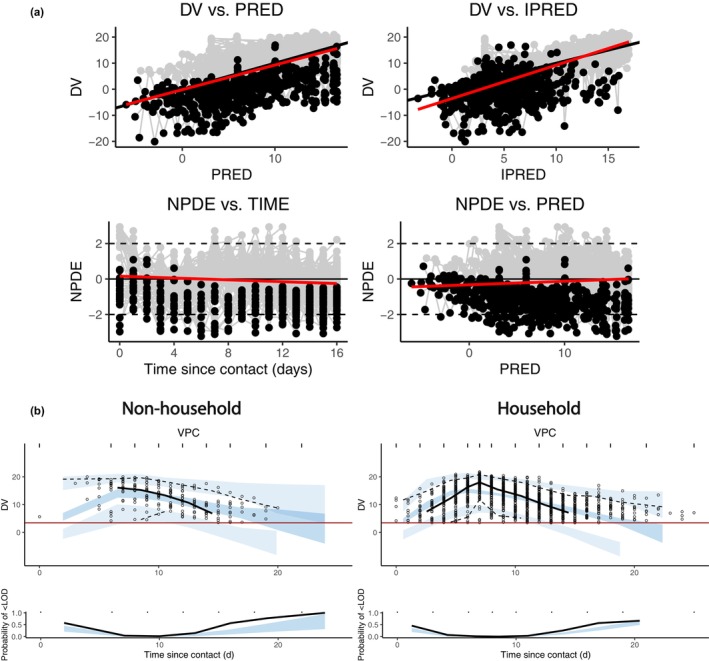
Graphic evaluation of the best fit model (TCLE model with immune response that increases viral clearance). (a) Goodness of fit plots. The black points are censored data, which indicate viral load below LOD. The black line is the expected trend, and the red line shows the mean of the plotted data. (b) VPCs for household and non‐household contacts. In the upper plot, black circles are observed viral loads. Simulated 90% predictions intervals of 5^th^, 50^th^, and 95^th^ percentiles of the data are shown as shaded bands with black lines showing corresponding percentiles of the observations. In the lower plot, the blue shaded area shows the 90% prediction interval of the model predicted proportion of samples below the LOD to compare with the observed proportion of viral loads below the LOD (black line). DV, dependent variable; IPRED, individual prediction; LOD, limit of detection; NPDE, Normalized Prediction Distribution Errors; PRED, population prediction; TCLE, TCL model with an eclipse phase; VPC, visual predictive check.

GOF plots and VPCs of the best fit model are shown in Figure 2. The plots showed that the model population and individual predictions are close to the observations and the residual errors mostly follow the distribution of $N\left(0,1\right)$. The predicted central trend and variability fitted the observed data better for non‐household patients than that for household contacts.

#### Parameter estimation

To deal with the problem of unidentifiability, some parameters in the model were fixed to literature values (Table 3). When the viral clearance c and productivity rate of infected cells k were fixed to 20, and 3 $\mathrm{da}y^{-1},$ ^13^ a better fit to the dataset was observed using the best fit structural model, compared to the case where these parameters were estimated. The impact of the fixed parameters was also tested with a sensitivity analysis (Supplements S4). When parameter c and k varies, the BICs were stable in most tested values, except when *k* was 1 $\mathrm{da}y^{-1}$ or 100 $\mathrm{da}y^{-1}$, and/or *c* was 1 $\mathrm{da}y^{-1}$.

**TABLE 3:** Estimated parameters of the best fit model when the viral clearance rate c, the eclipse rate k were either fixed or not.

Parameter (units)	Estimate	SE	RSE (%)	Estimate	SE	RSE (%)
*R*_0_	14.29	0.16	1.03	13.74	0.12	0.78
*δ* (day^−1^)	1.05	0.03	62.1	1.04	0.03	70.10
ϕ	0.21	0.15	9.75	0.24	0.17	5.44
θ	522	0.54	8.50	542.46	0.11	8.01
$V_{0}$ ($\text{copies}.mL^{-1}$)	27.9	0.16	4.90	23.33	0.48	7.70
ρ ($\text{copies}.\mathrm{cel}l^{-1}\;\mathrm{da}y^{-1}$)	6000 (fixed)	0.19	2.06	6000 (fixed)		
c ($\mathrm{da}y^{-1}$)	16.3	0.10	3.61	20 (fixed)		
k ($\mathrm{da}y^{-1}$)	3.29	0.09	7.33	3 (fixed)		
$d_{F}$ ($\mathrm{da}y^{-1}$)	0.4 (fixed)			0.4 (fixed)		
$T_{0}$	$1.33\times 10^{5}$ (fixed)			$1.33\times 10^{5}$ (fixed)		
BIC	12,328			12,182		

Abbreviations: BIC, Bayesian information criterion; δ, loss rate of infected cells; ϕ, maximal effect of the immune response; θ, level of F required to achieve 50% of the maximal effect; $V_{0}$, viral load at contact; ρ, viral replication rate; c, viral clearance rate; k, productivity rate of infected cells; $d_{F}$, elimination rate of immune response; RSE, relative standard error; $R_{0}$, basic reproductive number; SE, standard error; $T_{0}$, initial number of target cells.

In this case, the basic reproductive number, $R_{0}$, which is defined as $\frac{\rho \beta T_{0}}{\delta \left[c+\beta T_{0}+\phi F_{0}/\left(F_{0}+\theta \right)\right]}$, was estimated to be 13.36. Initial viral load was estimated to be 23.33 $\text{copiesm}L^{-1}$.

#### Covariate analysis

Covariate analysis was conducted on age, vaccination status, contact type (household/non‐household), type of variant, and symptomatic condition. Through plotting potential covariates versus viral load, only age showed some trend with viral load variability. Age was later added to the model as a covariate on β, δ, $V_{0}$, ϕ, and/or θ (Supplements S5). BIC decreased when age was added on δ, or $V_{0}$, or δ and $V_{0}$ (*p* < 0.05). Because the lowest BIC was achieved by adding age on δ, this model was selected for the simulation of the clinical trial.

### Simulation of antiviral therapies

#### Simulation of antiviral therapies varying in timing of administration and efficacy

**FIGURE 3:**
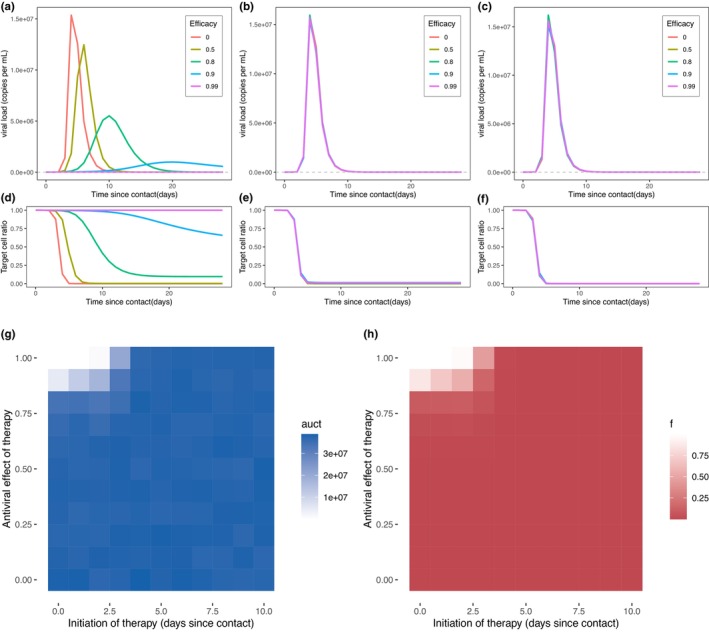
Predicted outcomes with therapies blocking de novo infection. Expected viral load trajectories with different treatment efficacies initiated 1 day (a), 4 days (b), and 7 days (c) after contact. Expected uninfected target cell proportion with different treatment efficacies initiated 1 day (d), 4 days (e), and 7 days (f) after contact. The red line which describes viral load in the case where efficacy is 0 indicates the occasion without treatment. The purple, blue, green, and mustard green lines are outcomes of treatments with 99%, 95%, 80%, and 50% antiviral efficacy, respectively. The gray horizontal line shows the limit of detection. (g) Viral load area under the curve (AUC) of blocking de novo therapies with different initiation times and efficacies. (h) Proportion of uninfected target cells with therapies with various initiation times and efficacies. Lighter colors represent a lower viral load AUC or a higher proportion of uninfected target cells, indicating a better therapeutic outcome.

Viral load AUC and fraction of uninfected target cells versus time since contact were considered in the case where antiviral therapies with viral inhibition efficacy of 99%, 90%, 80%, and 50% were administered at different timepoints post‐contact with an infectious case. This was compared with a control group. Treatment initiation time was set to 1 day (early intervention), 4 days (normal symptom onset time), and 7 days (mean viral load peak time) since contact.^31^ The simulated outcomes of antiviral therapy are shown in Figure 3. Administering the drug early after contact and increasing its efficacy, resulted in significant decrease of viral load AUC and increase of the proportion of cells prevented from infection. When treatment with an efficacy of 90% was initiated 1 day after contact, viral load AUC in patients was reduced by 69.9%, with most target cells (69.35%) kept uninfected 28 days after contact (Figure 3a,d). Delays in viral load peak were also observed, with higher efficacy of the therapy being associated to a later peak. Figure 3b,c,e,f show that when the therapy was initiated after day 4, there was little difference in the viral load AUC and fraction of uninfected target cells between the outcomes in the treated and control group. Even in the case where the efficacy was assumed to be as high as 90% in the simulation, the AUC in the treated group was 3.7 × 10^7^, close to the estimate in the control group (3.9 × 10^7^). The estimated fraction of target cells remaining uninfected was 0.4%, meaning that most target cells were infected 28 days after contact.

Heatmaps in Figure 3g,h show the effect of antivirals with efficacy ranging from 0% to 100% and initiation time from day 0 to day 10 since contact. Significant difference is observed only when therapy efficacy is higher than 80% and this is initiated within 3 days after contact. The therapeutic effect decreased with increase in days before the treatment was initiated. Hence, early antiviral treatment initiation and high drug efficacy are both important for the improvement of the therapeutic outcomes.

#### Comparison of the simulated outcome to the reported clinical trial data

**FIGURE 4:**
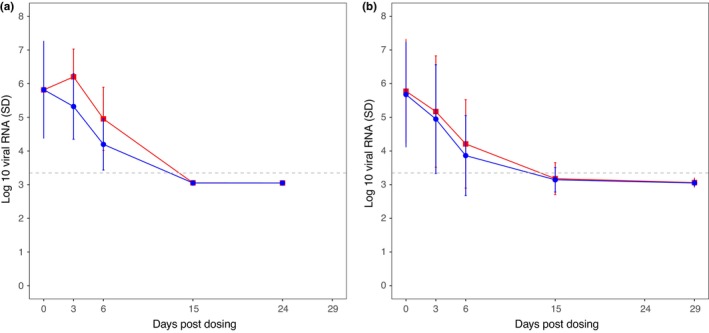
Comparison of the simulated and reported viral load trajectories in a clinical trial of tixagevimab‐cilgavimab. (a) The simulated and, (b) the reported viral load, in log_10_ scale. The blue line is the viral load after giving tixagevimab‐cilgavimab and the red line is the viral load in the placebo group. The data of clinical trial were digitized from Figure S1 in a published paper.^22^

Viral load trajectories in patients after receiving antiviral therapy or placebo were simulated and compared to the results from the actual phase III clinical trial of tixagevimab‐cilgavimab conducted by AstraZeneca. The simulated outcome showed a similar trend to the clinical trial outcome in terms of viral load reduction after treatment (Figure 4). A similar viral load at around 5.77 log_10_ copies per mL (588,843.7 copies per mL) was observed at treatment initiation. In both the treated and the placebo groups, the mean viral load reached a value below the LOQ at 12.7 days. In the simulated placebo group, a viral load peak was seen at around day 3, whereas in the clinical study the increase in viral load was not observed. High variability in viral load between patients was observed in both outcomes.

## DISCUSSION

In this study, a total of eight within‐host models of viral dynamics were tested on a single dataset, comparing their performance using standard graphic model evaluation as well as simulation‐based evaluation methods. Previous report on model comparison and selection based on the same dataset is limited.^16^

Here, the TCLE model with an immune response that increases viral clearance showed the best fit to the data. A different model was selected for SARS‐CoV‐2 modeling by Néant et al.,^14^ who reported a TCLE model with the immune responses increasing the clearance of productively infected cells as the best model. The discrepancy could be ascribed to the different datasets. In the community dataset considered in the present study, the samples were collected daily since contact regardless of symptomatology. Therefore, viral load data are available throughout the course of infection, whereas in Néant et al. and other studies viral load data were collected after symptom onset in the hospital.^14^, ^32^

The estimated reproductive number *R*_0_ with the best fit model was 13.74, which agrees with that reported in other studies (13.6).^33^ In early studies, $R_{0}$ was estimated to be much lower, ranging from 2.87 to 4.91.^12^, ^24^, ^34^ It was found that when considering viral dynamics earlier in the infection process, $R_{0}$ estimates are higher due to a higher infection rate β and a higher virus replication rate ρ at earlier stages.^12^ In this study, $R_{0}$ was calculated at the beginning of the infection (virus exposure) and not at symptom onset, resulting in a higher $R_{0}$. The estimated infected cell loss rate δ (1.04 $\mathrm{da}y^{-1}$) is similar to the numbers reported in previous studies (from 0.87 to 1.04 $\mathrm{da}y^{-1}$).^10^, ^12^, ^33^

The simulated drug effect in the present study is based on the mechanism of action of tixavegimab‐cilgavimab, which neutralizes the viruses before they enter host cells. The simulated outcome of tixavegimab‐cilgavimab in controlling viral replication was in a good agreement with the clinical trial data. Some discrepancies may be partly attributed to the different characteristics of the samples in the two datasets. The simulated outcomes also emphasized the importance of early intervention and high antiviral efficacy. Interestingly, if the efficacy was as high as 90%, the simulated peak viral load would be lower than $10^{6}$ copies/mL, which is the reported viral load threshold for in vitro viral culture.^35^, ^36^ The result indicates the possibility that by introducing an inhibiting viral entry drug early after contact, transmission of the virus could be stopped.

### Limitation

For household contacts, it is difficult to measure the time since infection, so the time of infection for household contacts were estimated by counting 7 days (mean time in the case of non‐household patients) before the viral load peak, leading to the difference in the simulated and observed viral load peak time (Figure 2).

The correlation between age and vaccination status was not included in the study. Considering that the elderly people are more vulnerable to the virus infection, vaccination among them were encouraged by the UK government. Therefore, the age of the vaccinated patients in the study was higher than that of the unvaccinated patients. The impact of vaccination might thus be indirectly related to the results.

The simulation is basic in terms of the assumed viral inhibition efficacy. The translation from common viral inhibition assay results, for example, half‐maximal effective concentration ($EC_{50}$) or plaque reduction neutralization test titers to the efficacy used in the simulation is not straightforward. A roughly 10 times drug concentrations higher than its $EC_{50}$ is reported to lead to efficacy of 90%.^14^ When discussing efficacy of therapy in this report, it only stands for the viral inhibition activity. However, the term is used differently in the clinical trial. Efficacy can be reported as a relative risk (RR) reduction in the incidence of developing severe disease or death. Although the viral load difference was not significant in the report, a high efficacy was observed in terms of RR reduction in severe COVID‐19 or death.^37^ This indicates the limitation of only using viral load as an evaluation on therapeutic effect of antiviral therapies.

## CONCLUSION

Among the tested eight models, the TCLE model with immune responses that enhance viral clearance showed the best fit to the data. Simulated antiviral outcome of tixagevimab‐cilgavimab emphasized the importance of early treatment and high antiviral efficacy. The simulated outcome showed a good agreement with clinical trial data in terms of viral load. This model could thus be further utilized for the simulation of hypothetical effects of new variants and be extended to incorporate and assess the potential impact of other antivirals with different mechanisms of action.

## AUTHOR CONTRIBUTIONS

S.Z., A.A.A., and C.H. wrote the manuscript. S.Z., A.A.A., C.H., and J.F.S. designed the research. S.Z. performed the research. S.Z., and C.H. analyzed the data.

## FUNDING INFORMATION

This study was part of a Master of Science in Pharmaceutics project at University College London. Funding for this work came from the UK Medical Research Council (MR/W015560/1).

## CONFLICT OF INTEREST STATEMENT

A.A.A. is currently an employee at GSK. All other authors declared no competing interests for this work.

## Supporting information


Data S1
