# Supplementary material for: SARS‐CoV‐2 viral dynamic modeling to inform model selection and timing and efficacy of antiviral therapy
Source: CPT Pharmacometrics Syst Pharmacol. 2023 Aug 21;12(10):1450–60. doi: 10.1002/psp4.13022 (PMC10583246; doi:10.1002/psp4.13022)
Supplement: Supplementary file 1 — Data S1 [file PSP4-12-1450-s001.pdf]

SUPPLEMENTARY MATERIAL: SARS-CoV-2 viral dynamic modelling to inform model selection and timing and efficacy of antiviral therapy

Table of Contents

Supplements S1 Differential Equations of the tested models 2

Supplements S2 Model code 4
   Model code 4
   Simulation code 7

Supplements S3 Graphic model evaluation on the tested models 12

Supplements S4 Sensitivity test on fixed parameters 21

Supplements S5 Covariate analysis 22

Supplements S1 Differential Equations of the tested models

M1 SI model

$$\frac{dV}{dt} = -\delta V$$

M2 TCL model

$$\frac{dT}{dt} = -\beta VT$$

$$\frac{dI}{dt} = \beta VT - \delta I$$

$$\frac{dV}{dt} = \rho I - cV$$

M3 rTCL model

$$\frac{df}{dt} = -\beta fV$$

$$\frac{dV}{dt} = \gamma fV - \delta V$$

M4 TCLE model

$$\frac{dT}{dt} = -\beta VT$$

$$\frac{dI_1}{dt} = \beta VT - kI_1$$

$$\frac{dI_2}{dt} = kI_1 - \delta I_2$$

$$\frac{dV}{dt} = \rho I_2 - cV - \beta VT$$

M5 TCLE + increasing cell refractory model

$$\frac{dT}{dt} = -\beta VT - \phi \frac{F}{F+\theta} T$$

$$\frac{dI_1}{dt} = \beta VT - kI_1$$

$$\frac{dI_2}{dt} = kI_1 - \delta I_2$$

$$\frac{dV}{dt} = \rho I_2 - cV - \beta VT$$

$$\frac{dF}{dt} = I_2 - d_f F$$

M6 TCLE + blocking cell infection model

$$\begin{aligned}
\frac{dT}{dt} &= -\beta\left(1-\phi\frac{F}{F+\theta}\right)VT \\
\frac{dI_1}{dt} &= \beta\left(1-\phi\frac{F}{F+\theta}\right)VT \ - kI_1 \\
\frac{dI_2}{dt} &= kI_1 - \delta I_2 \\
\frac{dV}{dt} &= \rho I_2 - cV - \beta VT \\
\frac{dF}{dt} &= I_2 - d_f F
\end{aligned}$$

M7 TCLE + increasing viral clearance model

$$\begin{aligned}
\frac{dT}{dt} &= -\beta VT \\
\frac{dI_1}{dt} &= \beta VT \ - kI_1 \\
\frac{dI_2}{dt} &= kI_1 - \delta I_2 \\
\frac{dV}{dt} &= \rho I_2 - cV - \beta VT - \phi\frac{F}{F+\theta}V \\
\frac{dF}{dt} &= I_2 - d_f F
\end{aligned}$$

M8 TCLE + promoting cytotoxicity model

$$\begin{aligned}
\frac{dT}{dt} &= -\beta VT \\
\frac{dI_1}{dt} &= \beta VT \ - kI_1 \\
\frac{dI_2}{dt} &= kI_1 - \delta I_2 - \phi\frac{F}{F+\theta}I_2 \\
\frac{dV}{dt} &= \rho I_2 - cV - \beta VT \\
\frac{dF}{dt} &= I_2 - d_f F
\end{aligned}$$

Supplements S2 Model code

Model code

```
rm(list=ls())
###### set working directory  ######
library(ggplot2)
library(nlmixr)

##### read data ######
vl_dat <- read.csv("20210920_ATACCC_L_ORF1ab Ct value measurements.csv")

##### viral load ######
# convert ct value to viral load
vl_dat$vl <- exp((37.933-vl_dat$CtT1)/1.418)*133.3333

# set the time of contact
# vl_dat2 <- orf1ab[complete.cases(orf1ab$DaysTrueExp2FirstSwab),]
# vl_dat2$Exp2Min <- vl_dat2$MinDay - 10 + vl_dat2$DaysTrueExp2FirstSwab
# mean(vl_dat2$Exp2Min) #7
vl_dat$time <- vl_dat$MinDay - 10 + vl_dat$TestDateIndex

for ( i in 1:nrow(vl_dat)){
  if(vl_dat[i,]$ContactType == "Household" & vl_dat[i,]$MinDay - 10 <= 7 ){
    vl_dat[i,]$time <- vl_dat[i,]$TestDateIndex + 7
  }
  else if(is.na(vl_dat[i,]$DaysTrueExp2FirstSwab) == FALSE){
    vl_dat[i,]$time <- vl_dat[i,]$MinDay-10 + vl_dat[i,]$DaysTrueExp2FirstSwab
                   + vl_dat[i,]$TestDateIndex
  }
  else{
    vl_dat[i,]$time <- vl_dat[i,]$MinDay - 10 + vl_dat[i,]$TestDateIndex
  }
}

#create numeric id number
vl_dat$id <- vl_dat$Subject
a<<-1
vl_dat$id2=1
for ( i in 2:nrow(vl_dat)){
  if (vl_dat[i,]$id!=vl_dat[i-1,]$id){
    a=a+1
    vl_dat[i,]$id2=a
  }
  else{
    vl_dat[i,]$id2=a
  }

}

vl_dat$id = as.integer(vl_dat$id2)
```

```
# set the dv into log scale
vl_dat$dv <- log(vl_dat$vl)

#set the cens and limit column
blod <-function(x){ if(x==40) {1}
  else if(x<40) {0}
}
vl_dat$cens <- sapply(vl_dat$CtT1,blod)

#limit column
vl_dat$limit <- (37.933-40)/1.418 + log(133.3333)
# make sure dv is set to LOD for all censored items
vl_dat$dv[vl_dat$cens == 1] <-  vl_dat$limit[vl_dat$cens == 1]
#vl_dat$limit[vl_dat$cens == 0] <- NA
# 2. set LIMIT close to 0 for CENS = 1
vl_dat$limit[vl_dat$cens == 1] <- log(0.001)

#EVID
vl_dat$EVID <- as.integer(0)

vl_dat$log_age <- log(vl_dat$Age/35)

############### RUN 7 ###############
# target cell limited model, F, Refractory
# age - delta
 TCL7<- function() {
  ini({
    tbeta  <- -16     # Rate constant for virus infection
    tdelta <- 0.112     # Death rate of infected cells
    trho   <- fix(log(6000))     # Viral replication
    tc     <- fix(log(20))       # Viral clearance
    tk     <- fix(log(3))       # productivity rate of infected cells
    tv0    <- 3.11       # Viral load at contact
    tdF    <- fix(log(0.4))
    tphi   <- -1.53
    ttheta <- 6.3

    #th_age <- -0.5
    t_age <- 1

    eta.beta ~ 0.01
    eta.delta ~ 0.01
    eta.v0 ~ 0.01
    eta.phi ~ 0.01
    eta.theta ~ 0.01

    add.err <- 1     # residual variability
  })
```

```
  model({
   beta  <- exp(tbeta + eta.beta)   # individual value of beta
   delta <- exp(tdelta + eta.delta + t_age*log_age)  #individual value of delta
   rho  <- exp(trho)      # individual value of rho
   c    <- exp(tc)         # individual value of c
   k    <- exp(tk)         # individual value of k
   v0  <- exp(tv0 + eta.v0)
   dF   <- exp(tdF)
   phi  <- exp(tphi + eta.phi)
   theta  <- exp(ttheta + eta.theta)

   A_T(0) = 1.33*10^5
   A_I1(0) = 0
   A_I2(0) = 1/30
   A_v(0) = v0
   FI(0) = 0

   d/dt(A_T) <- -beta * A_v * A_T
   d/dt(A_I1) <- beta * A_v * A_T - k * A_I1
   d/dt(A_I2) <- k * A_I1 - delta *  A_I2
   d/dt(A_v) <- rho*A_I2 - c*A_v - phi*FI/(FI + theta)*A_v - beta*A_v*A_T
   d/dt(FI) <-  A_I2 - dF * FI

   vt = log(A_v)
   vt ~ add(add.err)       # define error model
  })
}
# Check the model
nlmixr(TCL7)
#
run7.lst <- nlmixr(TCL7, vl_dat, est = "saem",
                   table = tableControl(npde = TRUE, censMethod = c("cdf")))

saveRDS(run7.lst, "F-viral clearance-m2.rds")
```

Simulation code

```r
rm(list=ls())
# Script to make predicted curves for different sites
library(ggplot2)
library(scales)
library("ggsci")
library(tidyverse)
library(deSolve)
library(dplyr)

########### simulations ########
## set up pf initial function, values need to be changed in function call
vl.sim.data <- function(tbeta = -14.65386721, ## beta from NM
                        tdelta = -0.02423612, ## delta from NM
                        trho = log(6000),
                        tc = log(20),
                        tk = log(3),
                        tv0 = 3.19332606, ## V0 from NM
                        tphi = -1.54922962,
                        ttheta = 6.28098060,
                        tdF = log(0.4),
                        ombeta = 0.01, ## IIV beta
                        omdelta = 0.01, ## IIV delta
                        omv0 = 0.01, ## IIV V0
                        omphi = 0.01, ## IIV Gamma
                        omtheta = 0.01,
                        nid = max(Sims$ID), ### pull in number of Ids in dummy population
                        age = Sims$age,
                        t_drug, ## day drug is started
                        t_tot = 28, ## total sim time
                        drug_eff = 0.99, ## drug theta from NM
                        Sims = Sims) ## simulated data frame
{
  # set simulation time frame based on preset total sim time
  times <- seq(0, t_tot + 1, 1)

  # drug indicator function
  drug_i <- matrix(ncol = 2, byrow = TRUE,
                   data = c(0, 0,
                            t_drug, 0,
                            t_drug + 1, 1,
                            t_tot +1, 1))
  # Linear interpolation function
  drug_input <- approxfun(x = drug_i[, 1], y = drug_i[, 2])
  # ode kim model
  model <- function (time, y, parms) {
    with(as.list(c(y, parms)), {
      indicator <- drug_input(time)
      drug_eff_t <- 1-drug_eff * indicator
      bet <- beta * drug_eff_t
```

```r
    # Derivatives
    dA_T <- -bet * A_v * A_T
    dA_I1 <- bet * A_v * A_T - k * A_I1
    dA_I2 <- k * A_I1 - delta *  A_I2
    dA_v <- rho  * A_I2 - c * A_v - phi * FI / (FI + theta) * A_v - bet*A_v*A_T
    dFI <-  A_I2 - dF * FI
    list(c(dA_T, dA_I1, dA_I2, dA_v, dFI))
  })
}

## set up population data frame to read in individual sims
out <- data.frame(rep(times, nid), sort(rep(c(1:nid), length(times))))
colnames(out) <- c("time", "nid")
out$pred <- NA

## simulate the Kim model for each ID in dummy population
for(i in c(1:nid)){
  # i <- 1
  # draw individual eta value from normal distribution
  ebeta  <- rnorm(n = 1, sd = sqrt(ombeta))
  edelta <- rnorm(n = 1, sd = sqrt(omdelta))
  ev0    <- rnorm(n = 1, sd = sqrt(omv0))
  ephi  <- rnorm(n = 1, sd = sqrt(omphi))
  etheta <- rnorm(n = 1, sd = sqrt(omtheta))
  #
  beta  <- exp(tbeta + ebeta)   # individual value of beta
  delta <- exp(tdelta + edelta + -0.11 * log(Sims$age[i]/35))  # individual value of delta
  rho  <- exp(trho)      # individual value of rho
  c    <- exp(tc)          # individual value of c
  k    <- exp(tk)          # individual value of k
  v0  <- exp(tv0 + ev0)
  dF   <- exp(tdF)
  phi  <- exp(tphi + ephi)
  theta  <- exp(ttheta + etheta)

  # set initial conditions and bind together individual parameters
  ini <- c(A_T = 1.33*10^5, A_I1 = 0, A_I2 = 1/30, A_v = v0, FI = 0)
  parms <- c(beta = beta, delta = delta, rho = rho, c = c, k = k, dF = dF, phi = phi, theta = theta)
  # make individual predictions
  pred <- data.frame(ode(ini, times = out$time[out$nid == i], model, parms))
  # read each individual into population data frame
  out$A_v[out$nid == i] <- pred$A_v
  out$A_T[out$nid == i] <- pred$A_T
}
out <- out[out$time <= t_tot, ]
out$pred[is.na(out$pred)] <- 1e-20
out$pred[out$pred == "NaN"] <- 1e-20  #
# add residual error
for(i in c(1:nrow(out))){
  out$pred[i] <- exp(rnorm(n = 1, mean = log(out$pred[i]), sd = 3))
```

```
  }
  out$pred[is.na(out$pred)] <- 1e-20
  out$pred[out$pred == "NaN"] <- 1e-20  #
  out$eff <- drug_eff
  out$days <- t_drug
  out
} # end of function

#### Simulation demographics data frame ####
age <- c(rep(18:65,2),65:75,75,75,76,77,78)
ID <- seq(1, length(age), 1)
Sims <- as.data.frame(cbind(ID, age))

############# simulation #########
# 1. make a placeholder for the results
out <- vl.sim.data(Sims = Sims, drug_eff = 1, t_drug = 3)[0,]
# 2. Loop through all individuals
for(i in c(1,4,7)){
  for(j in c(0,0.5,0.8,0.9,0.99)){
    out <- rbind(out, vl.sim.data(Sims = Sims,t_drug =i,drug_eff = j))
  }
}
# 3. write to a .csv
write.csv(out, "days.csv")

########### plot days ########
library(ggpubr)
out$f <- out$A_T/(1.33*10^5)

######## calculation function ##########
## Gives count, mean, standard deviation, standard error of the mean, and confidence interval (default 95%).
##   data: a data frame.
##   measurevar: the name of a column that contains the variable to be summariezed
##   groupvars: a vector containing names of columns that contain grouping variables
##   na.rm: a boolean that indicates whether to ignore NA's
##   conf.interval: the percent range of the confidence interval (default is 95%)
summarySE <- function(data=NULL, measurevar, groupvars=NULL, na.rm=FALSE,
                      conf.interval=.95, .drop=TRUE) {
  library(plyr)

  # calculate the length
  length2 <- function (x, na.rm=FALSE) {
    if (na.rm) sum(!is.na(x))
    else       length(x)
  }

  # calculate the length, mean and sd in each group
  # ddply , group_by + summarise in dplyr
  datac <- ddply(data, groupvars, .drop=.drop,
```

```
             .fun = function(xx, col) {
               c(N    = length2(xx[[col]], na.rm=na.rm),
                 mean = mean   (xx[[col]], na.rm=na.rm),
                 sd   = sd     (xx[[col]], na.rm=na.rm)
               )
             },
             measurevar
  )

  # rename
  datac <- plyr::rename(datac, c("mean" = measurevar))

  # calculate sd
  datac$se <- datac$sd / sqrt(datac$N)  # Calculate standard error of the mean

  # Confidence interval multiplier for standard error
  # Calculate t-statistic for confidence interval:
  # e.g., if conf.interval is .95, use .975 (above/below), and use df=N-1
  ciMult <- qt(conf.interval/2 + .5, datac$N-1)
  datac$ci <- datac$se * ciMult

  return(datac)
}

vl <- summarySE(out, measurevar="A_v",  groupvars=c("time", "eff", "days"))
f <- summarySE(out, measurevar="f",  groupvars=c("time", "eff", "days"))

#day1
vl1 <- vl[vl$days == 1,]
pl.1 <- ggplot(vl1) +
  theme_bw() +
  theme(legend.position = c(.95, .95),
        legend.justification = c("right", "top"),
        legend.box.background = element_rect(fill = "transparent"),
        panel.grid.major = element_blank(),
        panel.grid.minor = element_blank()) +
  labs(x = "Time since contact(days)", y = "viral load (copies per mL)", colour = "Efficacy") +
  geom_line(aes(x = time, y = A_v, group = eff, colour = as.factor(eff)), size = 1)+
  geom_hline(aes(yintercept = exp((37.933-40)/1.418)*133.3333), colour="grey", linetype="dashed")

f1 <- f[f$days == 1,]
pl.1f <- ggplot(f1) +
  theme_bw() +
  theme(legend.position = "none",panel.grid.major = element_blank(),
        panel.grid.minor = element_blank()) +
  labs(x = "Time since contact(days)", y = "Target cell ratio") +
  geom_line(aes(x = time, y = f, group = eff, colour = as.factor(eff)), size = 1)

#day4
vl2 <- vl[vl$days == 4,]
```

```
pl.2 <- ggplot(vl2) +
  theme_bw() +
  theme(legend.position = c(.95, .95),
        legend.justification = c("right", "top"),
        legend.box.background = element_rect(fill = "transparent"),
        panel.grid.major = element_blank(),
        panel.grid.minor = element_blank()) +
  labs(x = "Time since contact(days)", y = "viral load (copies per mL)", colour = "Efficacy") +
  geom_line(aes(x = time, y = A_v, group = eff, colour = as.factor(eff)), size = 1)+
  geom_hline(aes(yintercept = exp((37.933-40)/1.418)*133.3333), colour="grey", linetype="dashed")

f2 <- f[f$days == 4,]
pl.2f <- ggplot(f2) +
  theme_bw() +
  theme(legend.position = "none",panel.grid.major = element_blank(),
        panel.grid.minor = element_blank()) +
  labs(x = "Time since contact(days)", y = "Target cell ratio") +
  geom_line(aes(x = time, y = f, group = eff, colour = as.factor(eff)), size = 1)

#day7
vl3 <- vl[vl$days == 7,]
pl.3 <- ggplot(vl3) +
  theme_bw() +
  theme(legend.position = c(.95, .95),
        legend.justification = c("right", "top"),
        legend.box.background = element_rect(fill = "transparent"),
        panel.grid.major = element_blank(),
        panel.grid.minor = element_blank()) +
  labs(x = "Time since contact(days)", y = "viral load (copies per mL)", colour = "Efficacy") +
  geom_line(aes(x = time, y = A_v, group = eff, colour = as.factor(eff)), size = 1)+
  geom_hline(aes(yintercept = exp((37.933-40)/1.418)*133.3333), colour="grey", linetype="dashed")

f3 <- f[f$days == 7,]
pl.3f <- ggplot(f3) +
  theme_bw() +
  theme(legend.position = "none",panel.grid.major = element_blank(),
        panel.grid.minor = element_blank()) +
  labs(x = "Time since contact(days)", y = "Target cell ratio") +
  geom_line(aes(x = time, y = f, group = eff, colour = as.factor(eff)), size = 1)

pdf(file="unvacc.pdf",width = 14,height = 6)
ggarrange(pl.1, pl.2,pl.3,pl.1f,pl.2f,pl.3f,
          nrow = 2, ncol=3,heights = c(0.65, 0.35),labels = "AUTO")
dev.off()
```

Supplements S3 Graphic model evaluation on the tested models

Both goodness of fit plots and visual predictive check (VPC) were made to evaluate the performance of the models. In the goodness of fit plots, the black points are censored data, which indicate viral load under limit of quantification. The black line is the expected trend and the red line shows the mean of the plotted data. In the VPC plots, simulated 95% predictions intervals of 5th, 50th and 95th percentiles of the data are shown as shaded bands. The Lines are corresponding percentiles of the observations.

M1 SI model

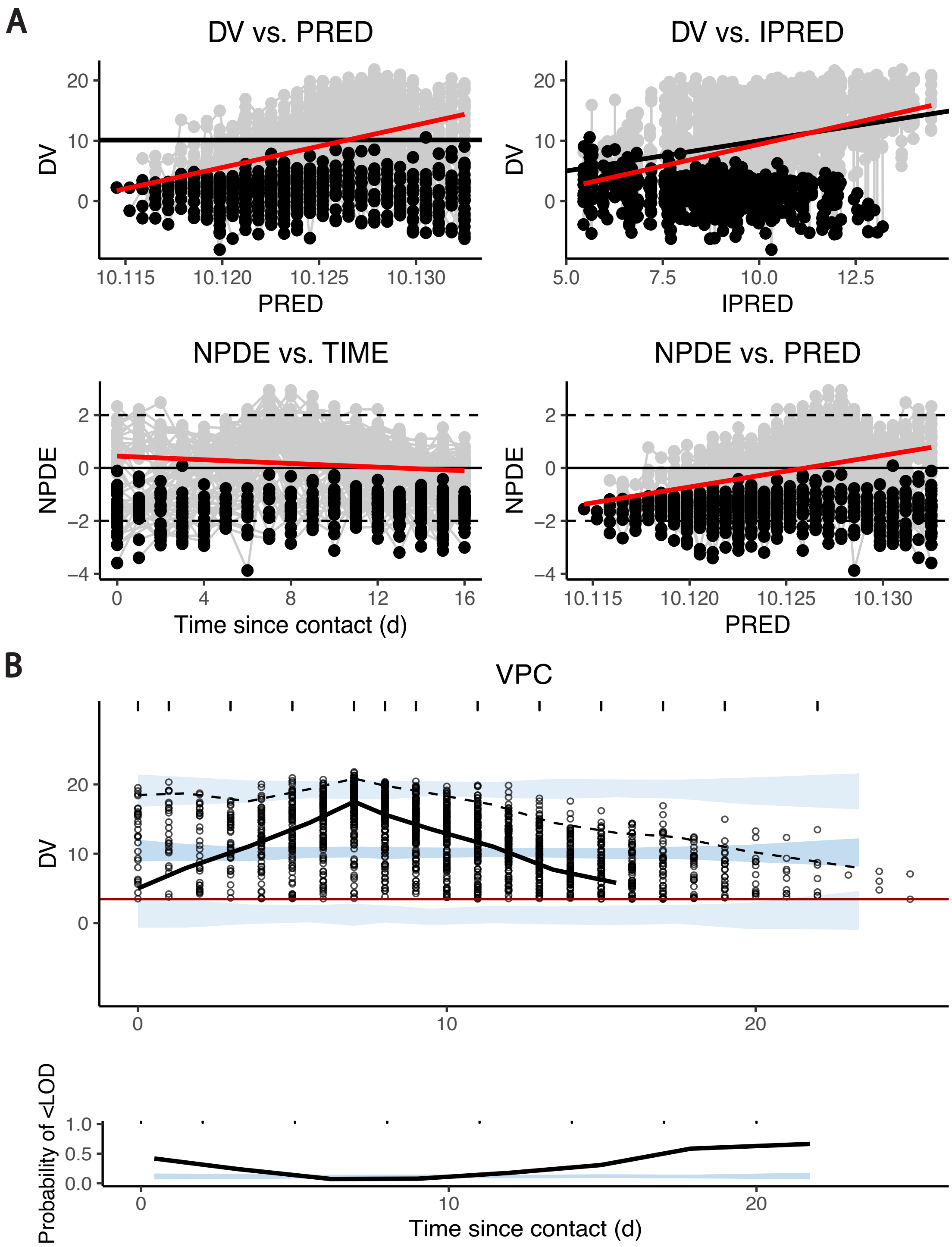

Graphic evaluation of M1 SI model. (A) Goodness of fit plots. (B) Visual predictive checks (VPC)

M2 TCL model

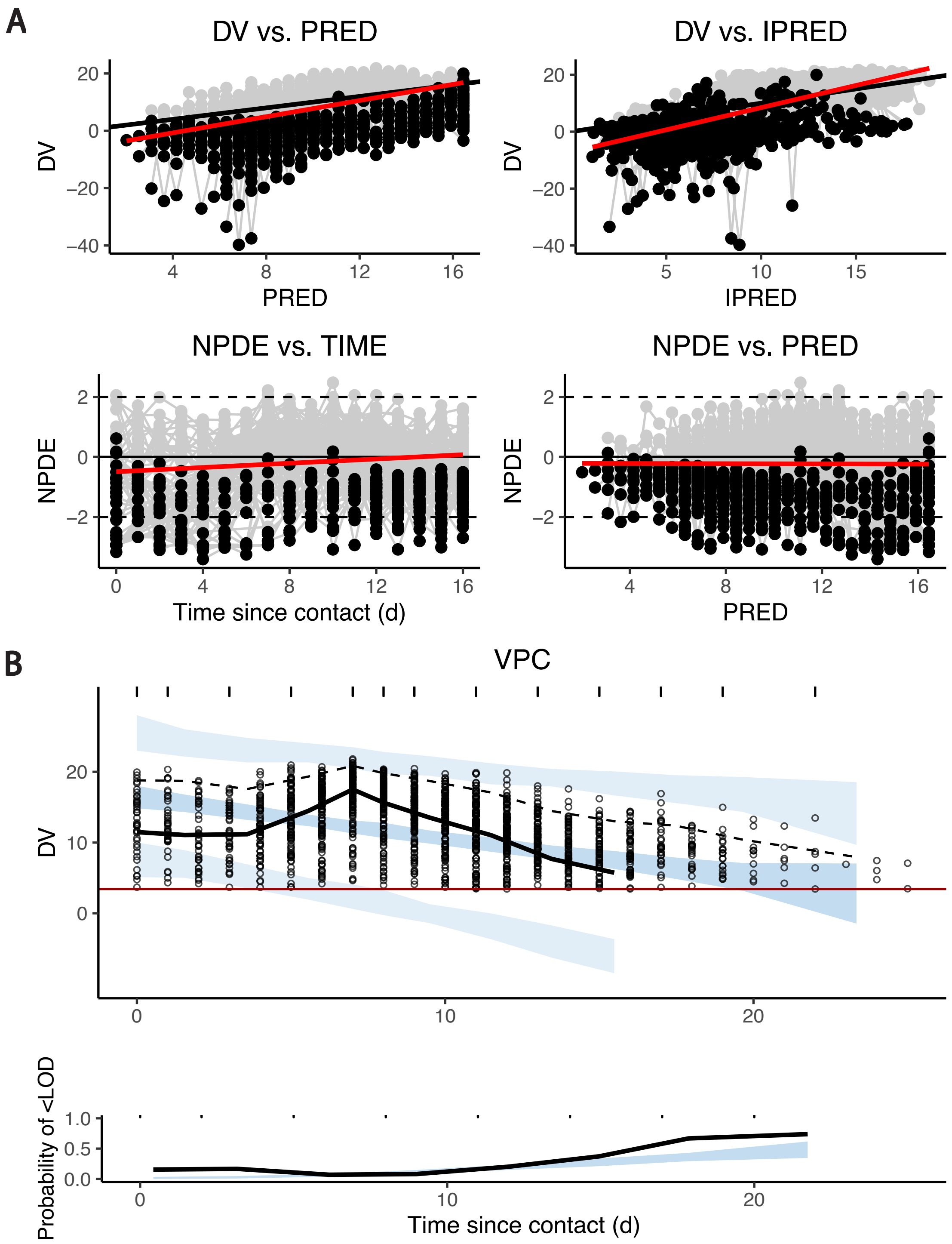

Graphic evaluation of M2 TCL model. (A) Goodness of fit plots. (B) Visual predictive checks (VPC)

M3 rTCL model

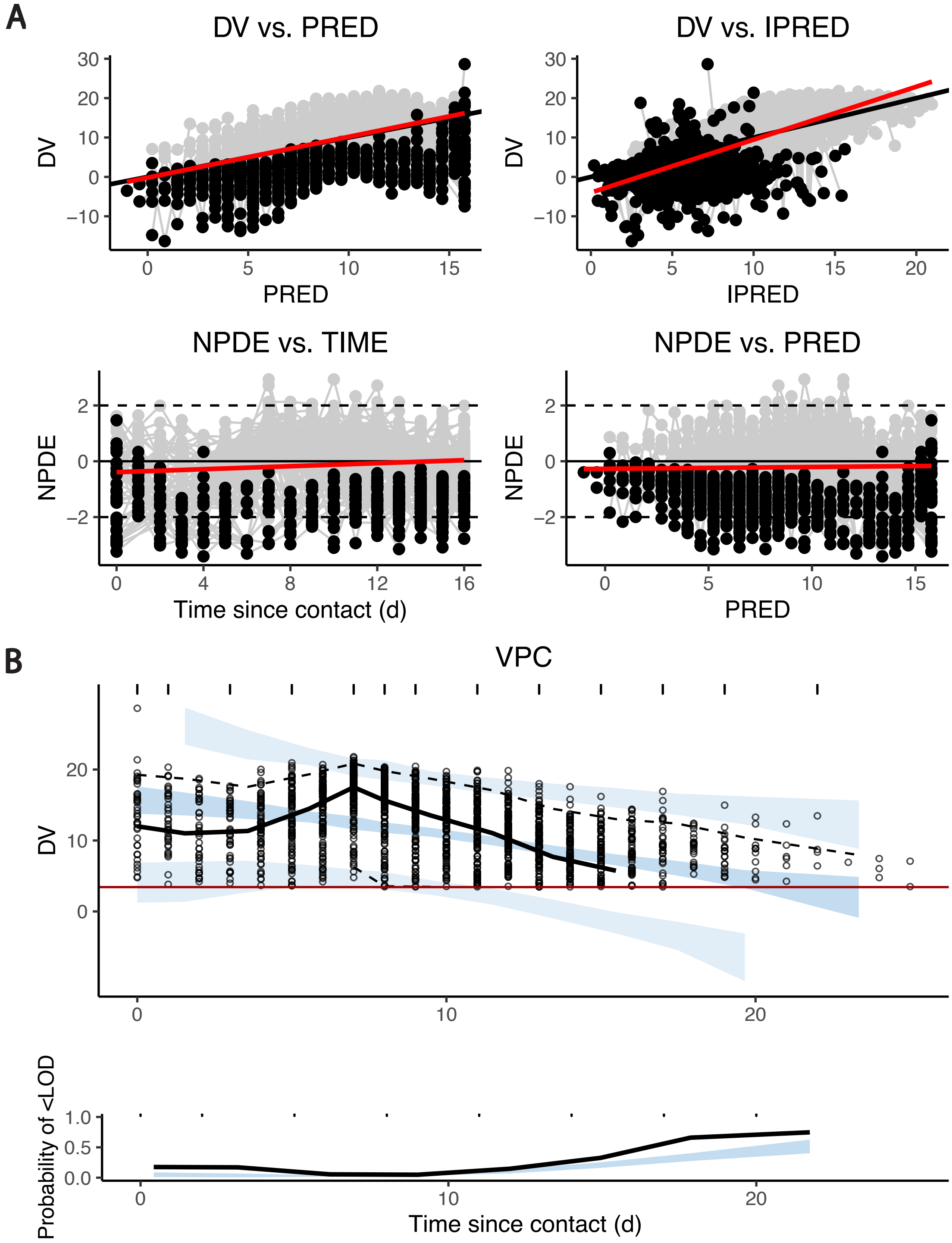

Graphic evaluation of M3 rTCL model. (A) Goodness of fit plots. (B) Visual predictive checks (VPC)

M4 TCLE model

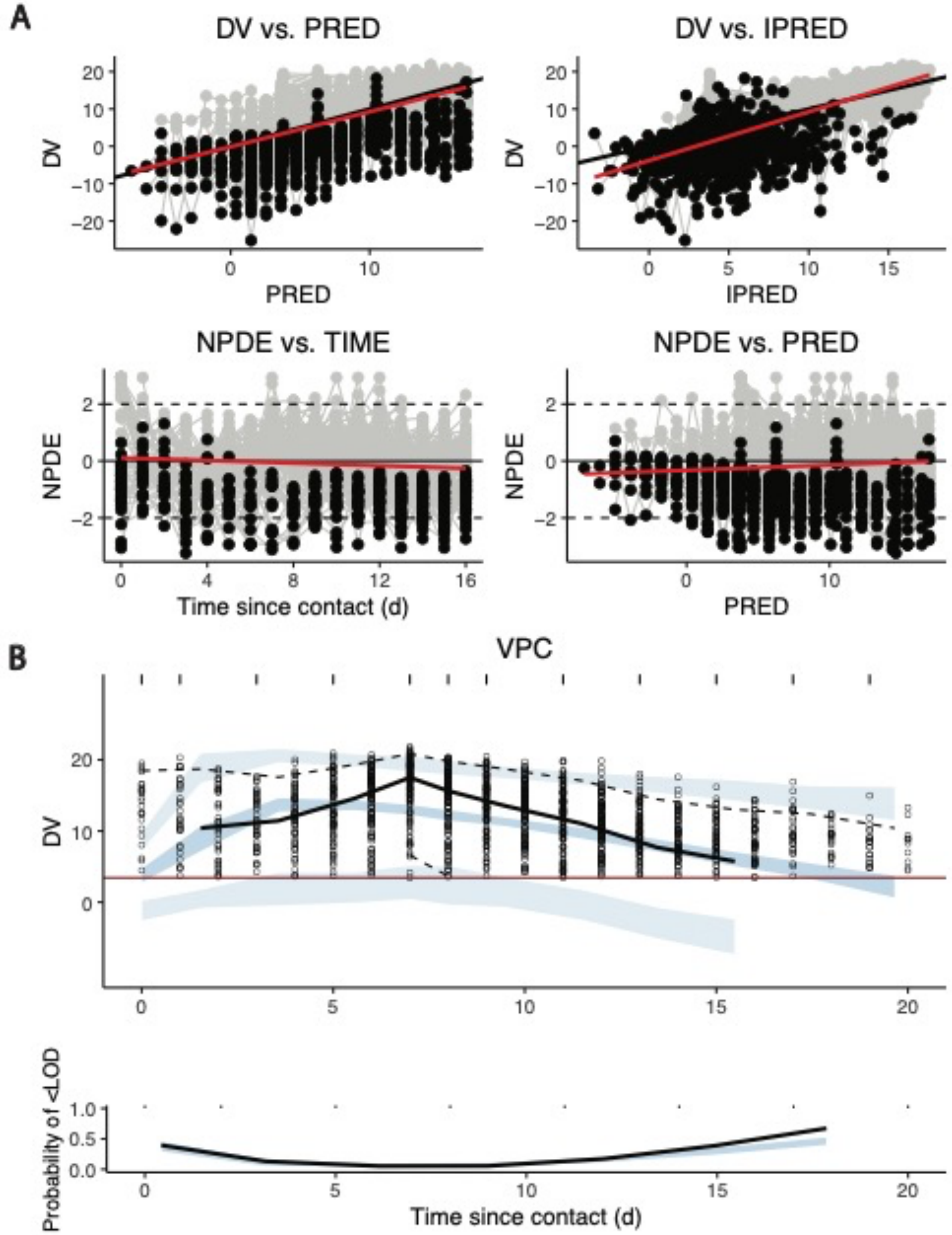

Graphic evaluation of M4 TCLE model. (A) Goodness of fit plots. (B) Visual predictive checks (VPC)

M5 TCLE + increasing cell refractory model

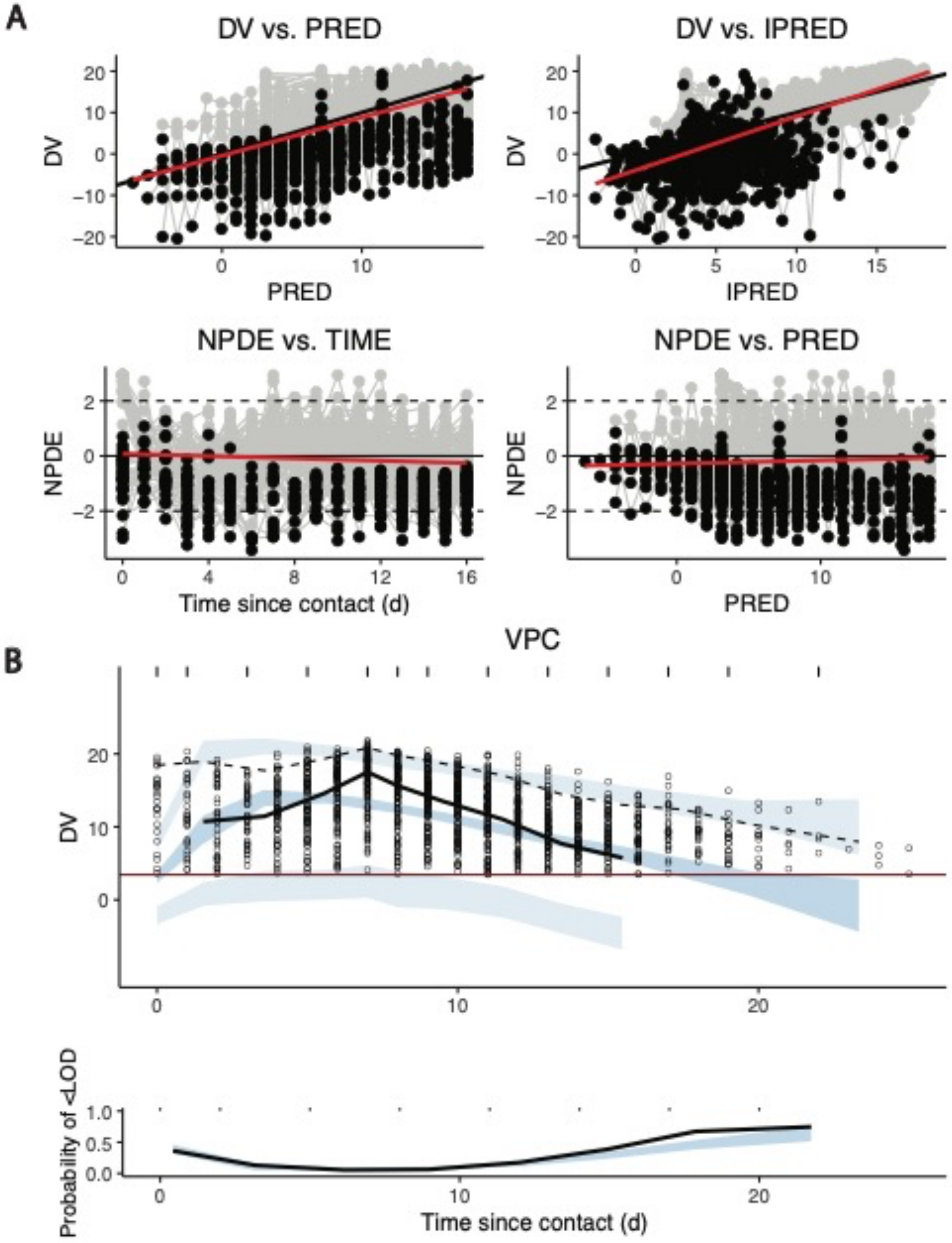

Graphic evaluation of M5 TCLE + increasing cell refractory model. (A) Goodness of fit plots. (B) Visual predictive checks (VPC)

M6 TCLE + blocking cell infection model

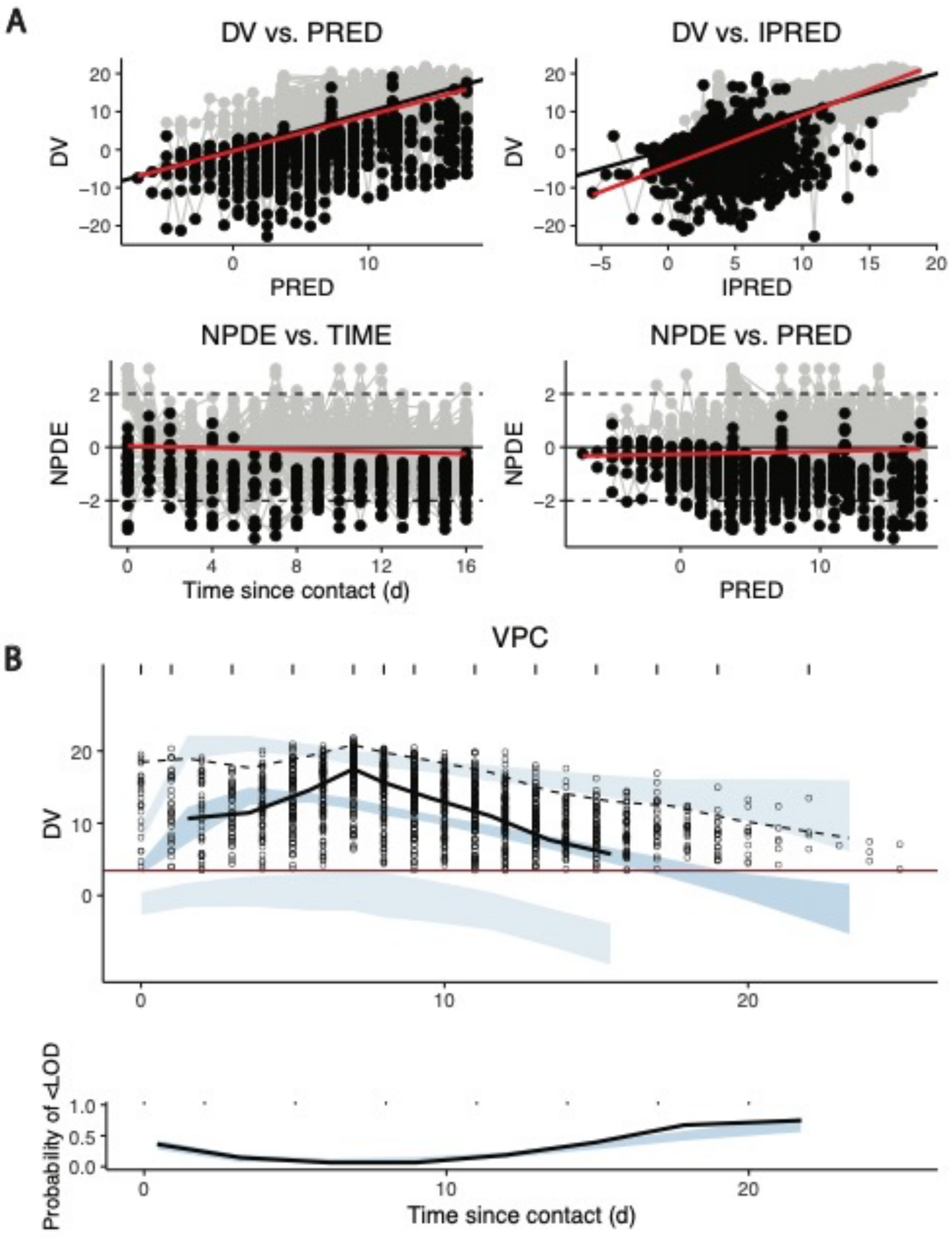

Graphic evaluation of M6 TCLE + blocking cell infection model. (A) Goodness of fit plots. (B) Visual predictive checks (VPC)

M7 TCLE + increasing viral clearance model

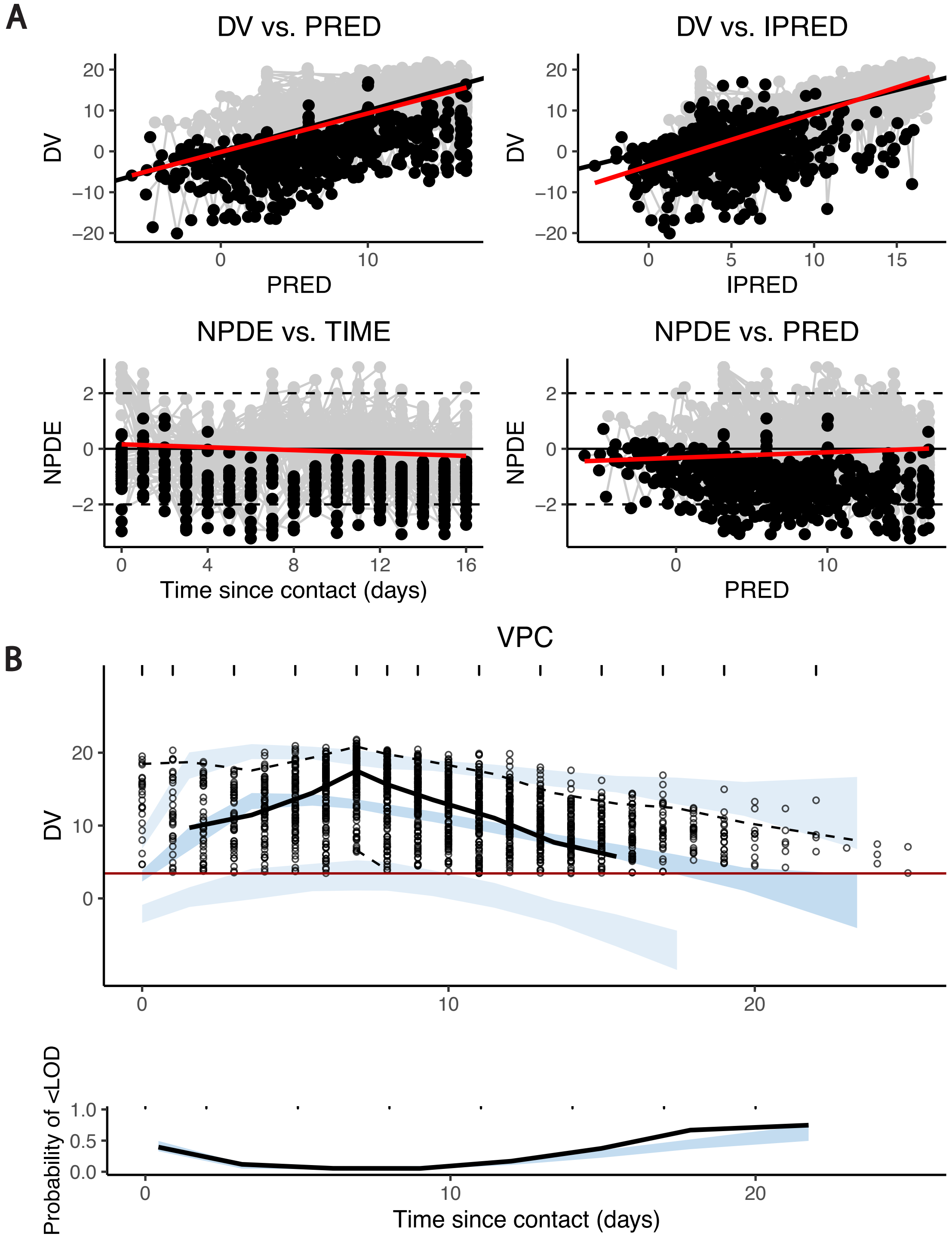

Graphic evaluation of M7 TCLE + increasing viral clearance model. (A) Goodness of fit plots. (B) Visual predictive checks (VPC)

M8 TCLE + promoting cytotoxicity model

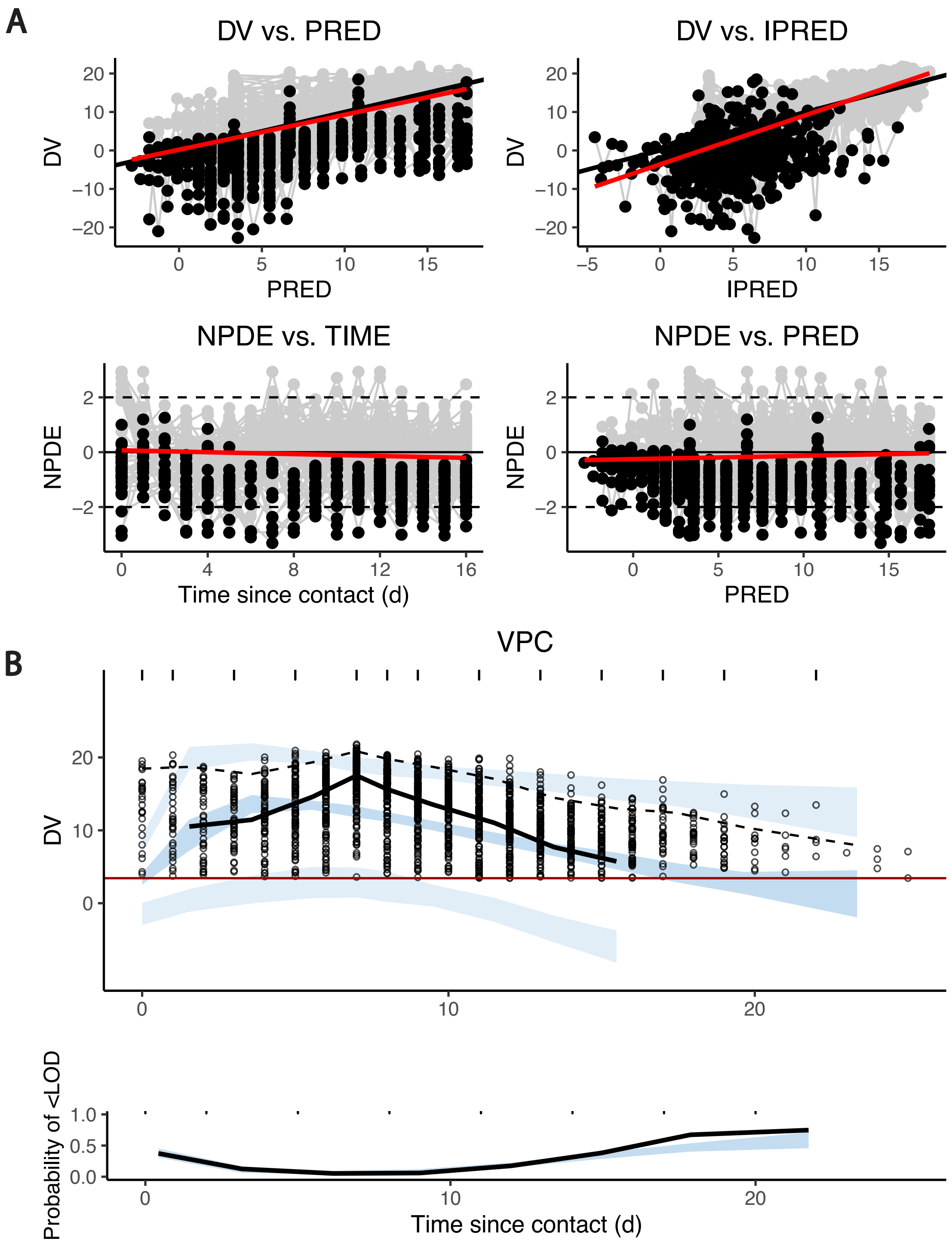

Graphic evaluation of M8 TCLE + promoting cytotoxicity model. (A) Goodness of fit plots. (B) Visual predictive checks (VPC)

Supplements S4 Sensitivity test on fixed parameters

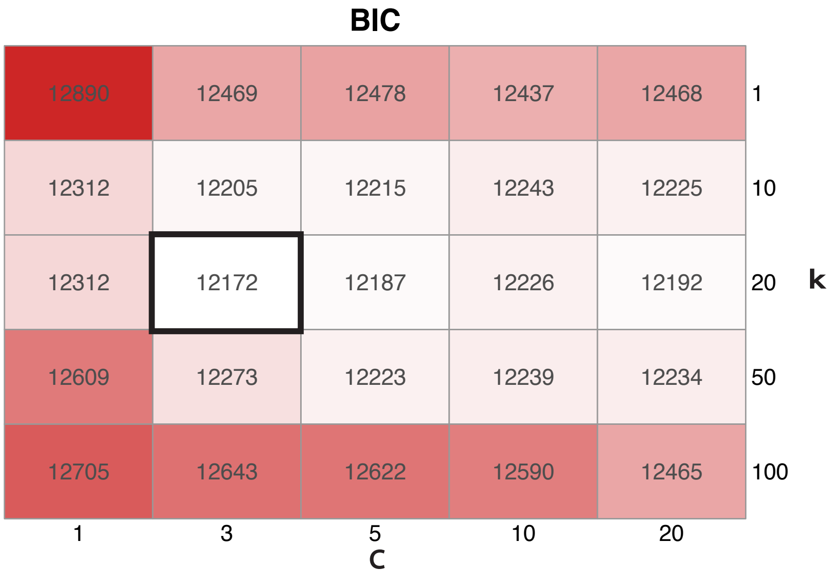

BIC was calculated with different values of the viral clearance rate c and the eclipse rate k. Dark red indicates larger values and white indicates lower value. The final model was chosen with k = 3 d^{-1} and c = 20 d^{-1} (black border).

Supplements S5 Covariate analysis

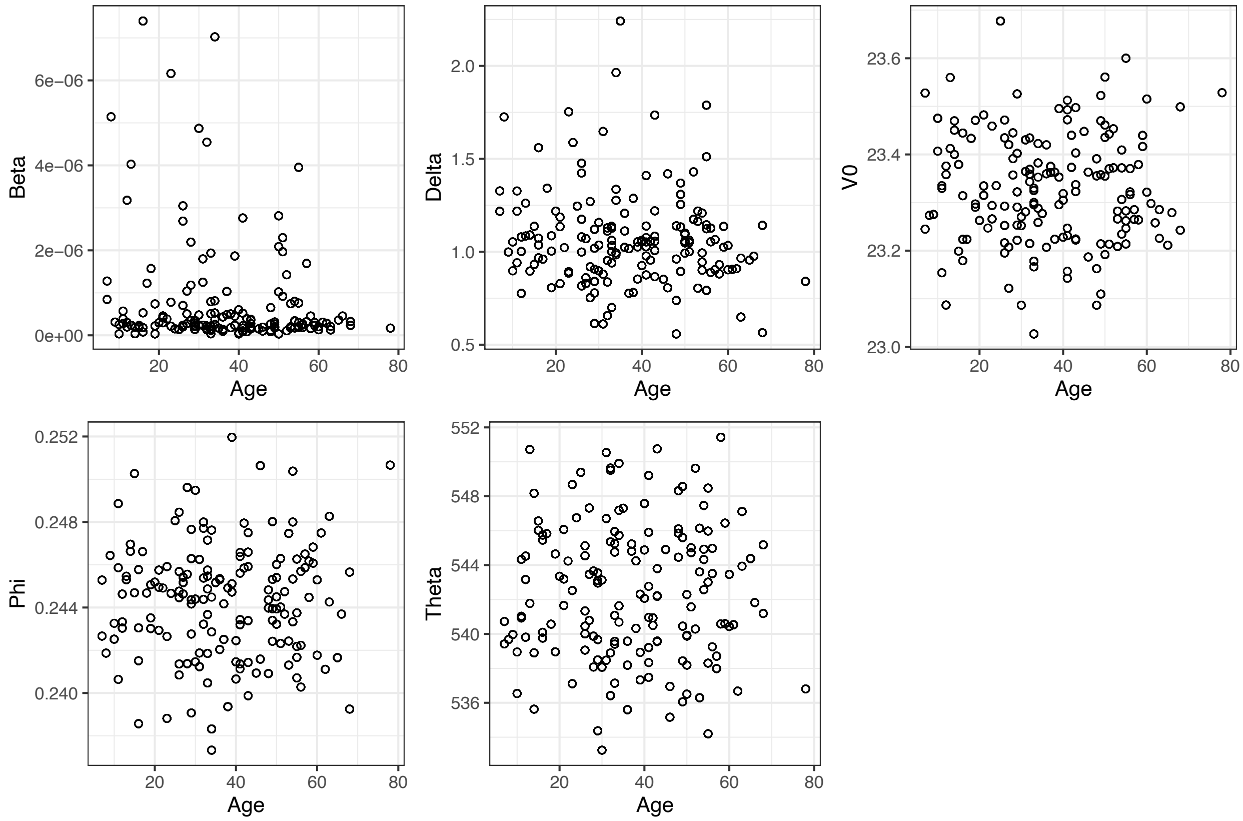

Graphic covariate analysis on age

Table 1. BIC of the best fit structural model with age added as a covariate on parameters

Variable	Base model	β	δ	V_0	ϕ	θ	δ, V_0
BIC	12182	7892	7874	7887	7894	7905	7887

BIC, Bayesian information criterion.
